# Body Composition Architecture and Injury Topology in Physically Active Young Adults: A Tanglegram-Based Cophylogenetic Approach

**DOI:** 10.3390/jcm15124678

**Published:** 2026-06-16

**Authors:** Jarosław Domaradzki

**Affiliations:** Department of Biological Principles of Physical Activity, Wroclaw University of Health and Sport Sciences, 51-612 Wrocław, Poland; jaroslaw.domaradzki@awf.wroc.pl

**Keywords:** sports injuries, body composition, training load, injury phenotypes, multidimensional analysis, hierarchical clustering, tanglegram, dbRDA

## Abstract

**Background/Objectives**: Injury occurrence in physically active young adults is considered a multifactorial phenomenon influenced by body composition and training-related characteristics. This study aimed to investigate the multidimensional relationships between body composition, training context variables, and injury phenotypes in physically active university students using exploratory multivariate approaches. **Methods**: The study included 418 physically active university students. Participants completed questionnaires regarding injury history, physical activity, and sport participation and underwent standardized anthropometric and body composition assessments. Analyses included Kendall’s Tau correlations, multiple correspondence analysis (MCA), hierarchical clustering, heatmap phenotyping, tanglegram-based clustering analysis, and distance-based redundancy analysis (dbRDA). The tanglegram analyses were intended as exploratory structure-matching procedures designed to evaluate similarities in hierarchical organization between domains rather than direct biological associations, causal relationships, or predictive effects. **Results**: Weak but significant associations were observed between selected body composition variables and injury outcomes, particularly for skeletal-muscle-related indicators and lower limb injuries. MCA and clustering analyses identified partially differentiated sport-training profiles and exploratory injury-burden phenotypes. Topology-based cross-domain matching analyses suggested partial structural correspondence between body composition, training context, and injury phenotypes; however, the most anatomically coherent patterns were observed for local body composition variables. Nevertheless, overall cross-domain concordance remained weak-to-moderate. dbRDA demonstrated statistically significant but weak associations for body composition (adjusted R^2^ = 0.027, *p* = 0.001) and the combined explanatory model (adjusted R^2^ = 0.022, *p* = 0.023), whereas the training context model was not significant (adjusted R^2^ = 0.002, *p* = 0.304). **Conclusions**: Injury occurrence was weakly associated with body composition and training context characteristics within a multidimensional exploratory framework. The findings are consistent with the interpretation of injury occurrence as a heterogeneous and predominantly multifactorial phenomenon and highlight the utility of multidimensional exploratory approaches for investigating complex injury-related patterns.

## 1. Introduction

Sports injuries represent a significant health and performance problem among physically active individuals and athletes. Contemporary conceptual models indicate that injury occurrence is not attributable to a single cause, but rather emerges from dynamic interactions between multiple intrinsic and extrinsic injury-related factors [1,2]. Early sports injury frameworks emphasized sequential prevention strategies and the identification of injury mechanisms, whereas subsequent theoretical developments highlighted the recursive and constantly changing nature of injury occurrence patterns [3]. These models suggest that repeated sport participation, adaptation, maladaptation, incomplete recovery, and previous injury history may contribute to variation in injury occurrence patterns over time.

Intrinsic determinants commonly include body composition, neuromuscular control, biomechanical characteristics, asymmetry, flexibility, and previous injury history, whereas extrinsic factors are primarily associated with training load, sport exposure, training intensity, environmental conditions, and workload progression [4,5,6]. Previous studies demonstrated that training-related variables such as excessive workload progression, cumulative exposure, inadequate recovery, and rapid changes in training intensity have been associated with injury occurrence in some populations [7,8]. At the same time, contemporary perspectives indicate that underloading and insufficient chronic training adaptation may also increase injury occurrence, emphasizing the complex and nonlinear nature of training–injury relationships [9,10]. 

Importantly, increasing evidence suggests that sports injuries should not be interpreted solely through isolated predictors or simple linear associations. Traditional reductionist approaches have been criticized for their limited ability to explain the multidimensional and context-dependent nature of injury occurrence [11]. Contemporary complex systems perspectives propose that injury occurrence may reflect interactions among interconnected biological, biomechanical, psychological, and training-related factors or profiles rather than single causal pathways [12]. Consequently, multidimensional analytical frameworks and pattern-recognition approaches may provide more ecologically valid insights into injury-related variability than traditional single-variable analyses.

Body composition has also been proposed as a factor associated with injury occurrence. Previous investigations demonstrated associations between adiposity, skeletal muscle mass, body asymmetry, and musculoskeletal injury occurrence, although findings remain inconsistent across different athletic populations and injury definitions [13]. Elevated body fat, altered lean mass distribution, and regional asymmetries have been associated with increased injury burden and lower extremity injuries in both athletic and military populations [14,15,16]. Similarly, longitudinal studies suggested that injury occurrence itself may unfavorably modify subsequent body composition trajectories, indicating potentially bidirectional relationships between morphology and injury processes [17].

In addition to isolated biological and morphological determinants, injury occurrence patterns may also depend on broader training context characteristics and sport participation profiles. Variables such as training volume, training frequency, cumulative exposure, sport specialization, contact exposure, physical activity level, and training experience may contribute to differences in the physiological and biomechanical demands experienced by physically active individuals [18,19]. Moreover, physically active university populations are characterized by heterogeneous sport participation profiles, including differences in sport type, training exposure, competitive level, technical specialization, and accumulated physical activity patterns. Such heterogeneity may contribute to distinct biomechanical and injury-related phenotypes that are not fully captured using traditional single-variable analytical approaches [20]. 

At the same time, the growing availability of multidimensional datasets has increased interest in exploratory analytical approaches capable of identifying hidden structures, phenotypes, and interaction patterns within complex injury-related data [21]. Multivariate profiling, clustering methods, dimensionality-reduction approaches, and machine-learning-oriented frameworks have increasingly been proposed as useful tools for identifying injury-related subgroups and multidimensional participation and injury profiles [22]. These approaches may be particularly relevant in physically active university populations characterized by heterogeneous training exposures, mixed sport participation profiles, and potentially weakly structured injury patterns.

Although previous studies have examined selected body composition characteristics, training-related factors, or injury outcomes separately, relatively few have investigated their multidimensional structural relationships using an integrated exploratory framework combining MCA, hierarchical clustering, heatmap phenotyping, tanglegram analysis, and dbRDA. Most previous studies have focused primarily on isolated injury-related factors, whereas considerably less attention has been directed toward the multidimensional structural organization linking morphological, training-related, and injury-related phenotypes.

The novelty of the present study lies in applying complementary multivariate exploratory approaches to examine injury occurrence as a heterogeneous and potentially weakly structured phenomenon within physically active young adults. This approach contributes to addressing a methodological gap related to the limited application of multidimensional structural analyses in sports injury research.

Therefore, the aim of this study was to investigate the multidimensional relationships between body composition, training context characteristics, and injury phenotypes in physically active university students. Specifically, the study aimed to: (1) examine associations between body composition, training-related variables, and injury outcomes; (2) identify multidimensional training-demographic and (3) participant-level injury phenotypes; (4) evaluate structural concordance between body composition, training context, and injury-related domains; and (5) determine which variables were most closely associated with the multidimensional organization of injury-related variability.

## 2. Materials and Methods

The present analysis combined data from two independently recruited cohorts of physically active university students assessed during consecutive data-collection periods (2022 and 2023). Both cohorts were recruited from the same university population and were evaluated using identical measurement procedures, eligibility criteria, and data-collection protocols. The cohorts were merged to increase sample size and enhance the stability of the multidimensional exploratory analyses. Prior to pooling, cohort comparability was evaluated across all study variables. Standardized mean differences were consistently below 0.10, indicating negligible between-cohort differences and supporting the pooling of both cohorts into a single analytical population.

### 2.1. Study Design

The study employed a cross-sectional design with retrospective assessment of injury history within a defined recall period using a standardized questionnaire. Participants were recruited through convenience sampling from physically active university students enrolled in physical education, sport, and physiotherapy programs at the Wroclaw University of Health and Sport Sciences during 2022–2023.

To increase sample size and facilitate multidimensional exploratory analyses, data from two independently recruited cohorts were pooled after confirming their comparability.

Participants completed an online questionnaire regarding injury history, physical activity, and training characteristics, followed by laboratory-based anthropometric and body composition assessments.

### 2.2. Ethics

The study protocol was approved by the Senate Research Ethics Committee of the Wroclaw University of Health and Sport Sciences (approval no. 13/2022) and conducted in accordance with the Declaration of Helsinki. Prior to participation, all students received detailed information regarding the study aims, procedures, and data handling principles, and provided electronic informed consent before data collection began.

### 2.3. Sample Size

The target sample size was determined based on methodological recommendations for multivariable epidemiological analyses. Because the study combined several exploratory multivariate approaches, including correspondence analysis, hierarchical clustering, tanglegram-based topology matching, and dbRDA, no single universally accepted a priori sample size calculation procedure was available. Therefore, the sample size rationale was based on general epidemiological recommendations intended to ensure sufficient representation of injury outcomes and adequate data structure for multivariate exploratory analyses within the analyzed dataset. Recommendations regarding the minimum number of outcome events have frequently been proposed in epidemiological research to ensure adequate representation of relatively infrequent outcomes and support multivariable analyses [23]. In the present study, these recommendations were used only as a general reference point for recruitment planning rather than as a formal sample size calculation for the specific exploratory multivariate analyses ultimately performed. Additionally, an a priori sample size calculation was performed using the standard proportion formula [24]:

$$n=(\frac{1.96}{\delta }{)}^{2}\times p\left(1-p\right).$$

Assuming a 95% confidence level, a margin of error of 0.05, and a conservative proportion estimate of 0.5, the minimum required sample size was estimated at 385 participants. To compensate for potential missing or incomplete data, the target sample was increased by 20%, resulting in an intended recruitment of approximately 460 participants. This sample size was considered sufficient to support sex-stratified analyses. In selected subgroup models, the recommended events-per-predictor criterion was treated as a flexible guideline rather than a strict requirement.

The final analytical sample (n = 418) exceeded the minimum sample size estimated from proportion-based calculations and was considered adequate for exploratory multivariate analyses aimed at exploring multidimensional structural patterns rather than developing predictive models.

### 2.4. Participants

A total of 454 university students participated in the study, including 219 men (48%) and 235 women (52%) recruited from Physical Education, Sport, and Physiotherapy programs at the Wroclaw University of Health and Sport Sciences during the 2022–2023 academic year. Participation was voluntary, and the sex distribution reflected the structure of these academic programs.

Eligible participants were physically active students regularly attending on-site classes. Students involved in university-level competitive sport or elite training programs were excluded to maintain comparability of habitual training exposure. Additional exclusion criteria included refusal to participate, incomplete key data, prolonged exemption from mandatory physical classes, and acute musculoskeletal injury within one month prior to assessment.

**Figure 1:**
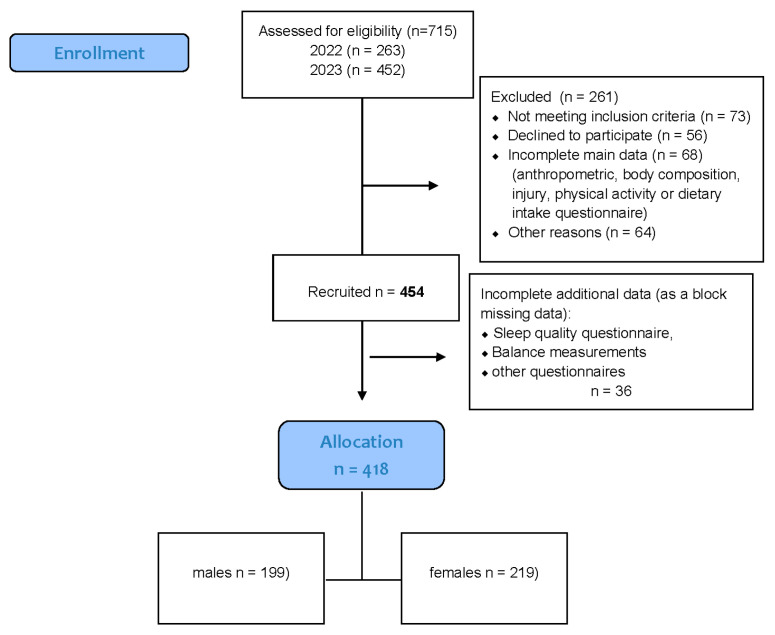
Flow diagram of the progress through all phases of data collection.

Among the 454 enrolled participants, 36 were excluded due to substantial missing data. The final analytical sample comprised 418 students. Mean physical activity levels exceeded the IPAQ criterion for high physical activity (≥3000 MET·min/week) in both men (3608 ± 1357) and women (3019 ± 1001). The participant selection process is illustrated in Figure 1.

### 2.5. Anthropometric Measurements

All anthropometric assessments were conducted under standardized laboratory conditions at the Biokinetics Research Laboratory, Wroclaw University of Health and Sport Sciences. Body height was measured to the nearest 0.1 cm using a GPM anthropometer (GPM Instruments GmbH, Susten, Switzerland). Body mass and body composition variables were assessed using bioelectrical impedance analysis (InBody Co., Ltd., Seoul, Republic of Korea), with measurements recorded to the nearest 0.1 kg.

Based on these measurements, Body Mass Index (BMI): BMI = body weight [kg]/body height^2^ [m^2^], Fat Mass Index (FMI): FMI = body fat [kg]/body height^2^ [m^2^], Skeletal Muscle Mass (SMI): SMI = skeletal muscle mass [kg]/body height^2^ [m^2^], and Fat-Free Mass Index (FFMI): FFMI = free-fat mass [kg]/body height^2^ [m^2^] were calculated. Additional body composition variables included bone mass (BM [kg]) and total body water (TBW [%]). Segmental muscle mass was assessed for the trunk, right and left upper limbs, and right and left lower limbs. 

Standardized absolute asymmetry (AA) indices were calculated for bilateral limb muscle mass measurements. The AA score reflects the magnitude of asymmetry without accounting for its direction and was calculated using the following formula [25]:

AA=(|R−L|)/(1/2×(R+L))×100

where AA—absolute asymmetry, R—right limb, L—left limb. 

According to Auerbach [26], AA values of 0–5% were classified as low asymmetry, 5–10% as moderate asymmetry, and >10% as high asymmetry. Elevated asymmetry values may indicate potential biomechanical imbalance and increased occurrence patterns of injury.

### 2.6. Questionnaire Measurements

#### 2.6.1. Injury Occurrence—Injury History Questionnaire (IHQ)

Musculoskeletal injury history during the previous 12 months was assessed using the standardized Injury History Questionnaire (IHQ), commonly applied in epidemiological studies of physically active populations [16]. Participants reported the number, type, and anatomical location of injuries related to sport, recreation, or daily activities, together with injury-related time loss. Consequently, the analyzed injury outcomes reflected overall musculoskeletal injury occurrence rather than exclusively sport-related injuries. For statistical analyses, injury occurrence was recoded into a binary variable indicating the presence (1) or absence (0) of injury. The number of injuries (n) was also included as an independent variable.

Injury location variables were additionally classified into four anatomical regions: trunk (with head and neck), upper limbs, and lower limbs. For each anatomical region, both the total number of reported injuries and a binary occurrence variable were determined, where 0 indicated no injury and 1 indicated the occurrence of at least one injury within a given body region during the observation period.

Similarly, injury type variables were categorized according to the predominant injury mechanism and tissue involvement. The analyzed injury types included: joint sprain, bone fracture, muscle strain, and skin abrasion. For each injury type, the total number of reported injuries was calculated, and corresponding binary occurrence variables were created (0 = no injury; 1 = at least one injury of a given type).

#### 2.6.2. Subsequent Injury Classification

Participants reporting at least one injury additionally indicated whether the injury involved the same anatomical location and/or injury type as a previous injury. Based on these responses, subsequent injuries were classified as recurrent (same location and same type), related (same location or same type only), or unrelated (different location and different type). Due to limited subgroup counts, related and unrelated injuries were combined into a single category (“other subsequent injury”) for statistical analyses.

#### 2.6.3. Total Physical Activity (TPA)—International Physical Activity Questionnaire (IPAQ)

Physical activity was assessed using the Polish version of the International Physical Activity Questionnaire—Long Form (IPAQ-LF) [27], administered online via Google Forms. The primary variable used in the analyses was total physical activity (TPA; MET·min/week).

#### 2.6.4. Training Volume (TV) and Training Age (TA)

Training volume was operationally defined as the total weekly duration of sport-specific training expressed in hours per week (h·week^−1^). In contrast to comprehensive training load models that additionally incorporate exercise intensity, the present classification was based exclusively on weekly training duration as a practical indicator of accumulated training exposure. Participants were categorized into four training volume groups: low volume/beginner (1–3 h·week^−1^), recreational/fitness (4–7 h·week^−1^), advanced amateur/serious competitor (8–15 h·week^−1^), and elite/high-volume training (>15 h·week^−1^), adapted from previously published sport participation frameworks [28].

Training age (also referred to as training experience) was defined as the cumulative duration of systematic sport-specific training expressed in years. Unlike chronological age, training age may reflect long-term exposure to organized training and accumulated sport-specific experience and is commonly used as an indicator of athletic development and training status. Based on established sport science and strength-and-conditioning frameworks, participants were categorized into four training age groups: beginner/novice (<2 years), intermediate (2–5 years), advanced (5–8 years), and elite/highly experienced (>8 years) [29].

#### 2.6.5. Sports Discipline Classification According to Biomechanical and Physiological Characteristics

Sports disciplines reported by participants were classified into broader functional categories according to their predominant movement characteristics, physiological demands, and typical training structure. The classification included: (1) team sports, characterized by intermittent high-intensity efforts, multidirectional movement, and frequent player interaction; (2) combat sports, involving direct opponent confrontation, high neuromuscular demands, and asymmetrical movement patterns; (3) endurance sports, dominated by cyclic and prolonged aerobic activity; (4) strength/power sports, primarily focused on resistance-based or high-force training stimuli; and (5) technical/coordination sports, emphasizing precision, motor control, flexibility, coordination, or aesthetic movement execution. Team sports included football, basketball, volleyball, handball, and frisbee. Endurance sports comprised running, cycling, swimming, triathlon, and track and field disciplines. Combat sports included kickboxing, Muay Thai, and taekwondo. Strength/fitness activities included gym-based resistance training and fitness training. Technical/coordinative disciplines included dance, tennis, table tennis, acrobatics, badminton, parkour, yoga, archery, and climbing.

Additionally, two supplementary sport participation classifications were established to better characterize the training and injury-related context of the participants.

First, disciplines were categorized according to contact exposure (contact vs. non-contact). The contact category included sports characterized by direct physical interaction between players or opponents, frequent collisions, tackling, grappling, or interpersonal contact. This group comprised football/soccer, basketball, volleyball, handball, baseball, ultimate frisbee, karate, judo, taekwondo, kickboxing, boxing, Muay Thai, fencing, and other combat sports. In contrast, the non-contact category included disciplines primarily based on individual movement execution, cyclic locomotion, technical precision, or resistance-based exercise without direct bodily contact between participants.

Second, sports were classified according to participation structure (individual vs. team). The team category included sports performed collectively with coordinated interaction between multiple players, such as football/soccer, basketball, volleyball, handball, baseball, and ultimate frisbee. The individual category included all remaining disciplines, in which performance primarily depended on individual physical, technical, or physiological capacity rather than direct team cooperation.

### 2.7. Handling Missing Data

A small number of missing observations were identified during the initial data screening process. As illustrated in the participant selection flowchart (Figure 1), individuals with incomplete data for the primary analytical variables, including body composition, injury-related, and training context measures, were excluded prior to dataset construction.

Consequently, the final analytical dataset consisted exclusively of complete cases for all variables included in the statistical analyses, and no imputation procedures were applied.

### 2.8. Statistics

**Figure 2:**
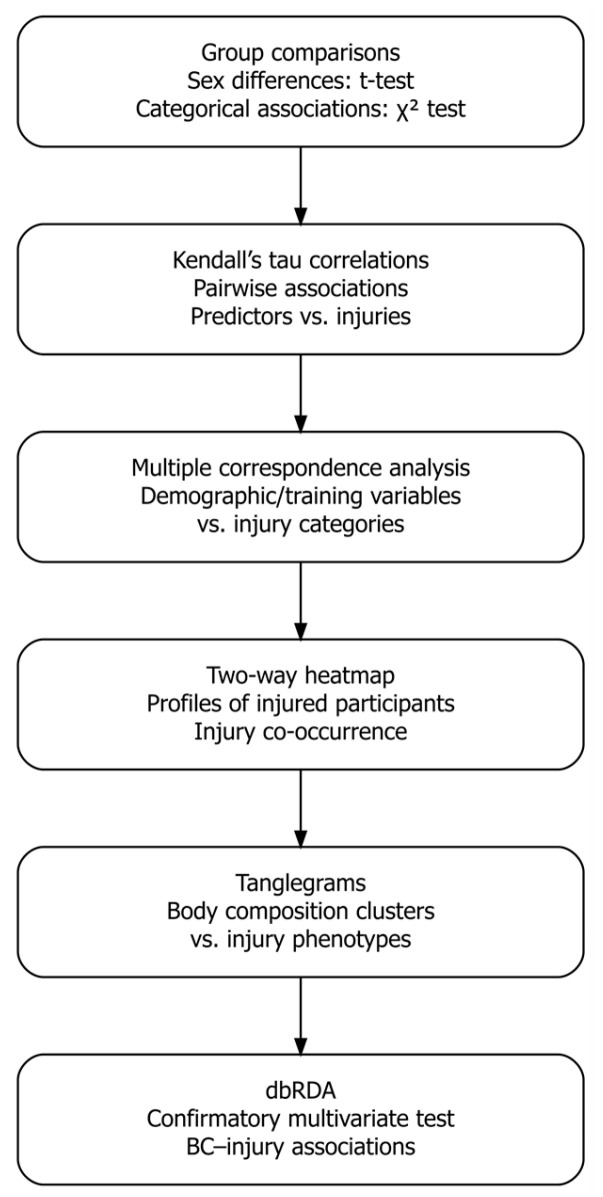
Analytical workflow summarizing the sequential statistical procedures applied in the study, including preliminary group comparisons, Kendall’s tau correlations, multiple correspondence analysis, two-way heatmap profiling, tanglegram-based clustering of body composition and injury phenotypes, and confirmatory distance-based redundancy analysis (dbRDA).

Distributional assumptions for continuous variables were checked with the Shapiro–Wilk test, while homogeneity of variance was assessed using Levene’s test. Descriptive statistics are presented as means ± standard deviations with 95% confidence intervals or as frequencies and percentages, as appropriate. The overall analytical strategy is summarized in Figure 2.

#### 2.8.1. Descriptive Statistics and Simple Comparisons—Statistical Characteristics of the Participants

Sex-related differences were assessed using independent-samples *t*-tests for continuous variables and chi-square tests for categorical variables.

#### 2.8.2. Kendall’s Tau Correlations—Relationship Screening

Kendall’s tau correlation analysis was applied to assess monotonic associations between injury-related variables and selected demographic, training context, and body composition parameters. Owing to the presence of non-normal distributions and ordinal or binary variables, Kendall’s tau was selected as a robust nonparametric measure of association strength and direction.

Because the Kendall’s tau analyses were exploratory and intended primarily to identify potential association patterns rather than to perform formal hypothesis testing, no adjustment for multiple comparisons was applied. Consequently, statistically significant associations should be interpreted cautiously and considered hypothesis-generating rather than confirmatory findings.

#### 2.8.3. Multivariate Correspondence Analysis—Analysis of the Training–Demographic Context

Multiple correspondence analysis (MCA) was performed to explore relationships among categorical variables, including sex and training-related characteristics. The method enabled visualization of multivariate association patterns and facilitated the identification of proximities among participant and injury-related profiles within a reduced-dimensionality space. Because body composition and injury occurrence may vary across demographic and training context characteristics, an exploratory MCA was performed to characterize the multidimensional organization of participant profiles prior to conducting injury-focused multivariate analyses.

#### 2.8.4. Two-Way Heatmap—Visualization of Participant-Level Injury Profiles

Two-way heatmap analysis was used to explore participant-level injury profiles and patterns of injury co-occurrence. The analysis additionally enabled assessment of whether injury distributions differed according to sex, thereby informing the decision on whether men and women should be analyzed jointly or separately in subsequent analyses.

Two-way clustered heatmaps were used to visualize multidimensional injury profile patterns and explore potential co-occurrence structures among injury locations and injury types across participants.

#### 2.8.5. Tanglegrams and Topology-Based Cross-Domain Matching with Cophenetic Statistics

Tanglegram and cophylogenetic analyses were applied to visualize structural similarities among body composition, training context, and injury-related phenotypes. Separate hierarchical dendrograms were generated for global body composition, local body composition, training context variables, and injury profiles using Euclidean distance matrices derived from standardized variable profiles and Ward.D2 hierarchical clustering.

To identify cross-domain pairings between domains, the Hungarian algorithm (linear sum assignment problem) was applied to match variables according to minimal Euclidean distances between multidimensional variable profiles. Lower Euclidean distances indicated greater profile similarity between paired variables across participants. For training context analyses, categorical variables were transformed into binary dummy indicators prior to standardization and clustering procedures.

Cross-domain links were subsequently visualized within tanglegrams using line thickness and color intensity to represent relative similarity categories (higher, intermediate, and lower similarity). To improve figure readability, only the most prominent and intermediate similarity links identified by the matching procedure were highlighted in the final visualizations, whereas weaker links were deemphasized or omitted from graphical presentation. This visualization step did not affect the underlying matching procedure, dendrogram structure, or concordance statistics, which were generated algorithmically.

Whereas clustering and ordination methods describe structures within a single analytical domain, the tanglegram framework was used to evaluate similarities in hierarchical organization across independently derived domains. This approach enabled assessment of whether body composition, training context, and injury-related variables exhibited comparable multidimensional structures beyond pairwise associations or within-domain clustering patterns.

To evaluate dendrogram fidelity, cophenetic correlation coefficients (CCC) were calculated separately for each hierarchical tree. Additionally, link-level cophenetic concordance analyses based on Spearman’s rho and Kendall’s tau were used to assess topological agreement between paired cross-domain structures.

#### 2.8.6. Distance-Based Redundancy Analysis (dbRDA)—Constrained Multivariate Explanation

Distance-based redundancy analysis (dbRDA) was applied as a complementary multivariate approach to examine associations between body composition profiles and injury-related variables. The analysis was based on Gower distance matrices, allowing the simultaneous inclusion of continuous, binary, and categorical variables.

dbRDA enabled evaluation of the extent to which variability in injury topology was associated with global and local body composition characteristics. The method additionally provided ordination plots illustrating multivariate relationships among participants, body composition variables, and injury phenotypes.

The analysis produced pseudo-F statistics, proportions of constrained variance (R^2^), and permutation-based *p*-values, which were used to assess the statistical significance and magnitude of multivariate associations. Higher R^2^ values indicated a greater proportion of injury pattern variability accounted for by the constrained ordination model, whereas significant permutation tests were interpreted as evidence of non-random multivariate structure within the analyzed data.

All analyses were conducted in Statistica 14.0 (TIBCO Software Inc., Palo Alto, CA, USA) and RStudio (2025.09). Statistical significance was set at *p* < 0.05.

### 2.9. AI Transparency Statement

Generative artificial intelligence (AI) tools were used in accordance with COPE and MDPI transparency recommendations and were limited to preparatory, technical, and editorial support during manuscript development. Chat Academia (v.1.0, 2025), Elicit (v.2.0, 2025), and SciSpace (2025) were used to support literature exploration, manuscript organization, and refinement of methodological terminology. In selected technical instances, Julius AI (2025) and ChatGPT (OpenAI, GPT-4.1, 2025) were used to assist with troubleshooting and clarification of R programming procedures and draft code syntax. AI tools did not contribute to the study design, data collection, statistical decision-making, or interpretation of results. All AI-assisted outputs were manually reviewed, independently verified, and modified where necessary. The authors assume full responsibility for the integrity and final content of the manuscript.

## 3. Results

### 3.1. General Characteristics of the Study Sample

Baseline characteristics are summarized in Table 1. Data are reported as mean ± standard deviation with 95% confidence intervals. Anthropometric parameters differed significantly by sex, with males showing higher values across all somatic measures (all *p* < 0.05). In addition, males reported significantly higher physical activity levels (TPA) and training age (TA) than females (both *p* < 0.001), but not training volume (TV) (*p* = 0.200). Males presented more lower limb asymmetry (*p* < 0.001), while females presented more upper limb asymmetry (*p* < 0.001). 

Across the entire cohort (N = 418), 51.4% of participants reported experiencing at least one injury within the preceding 12 months. Injury occurrence was significantly more frequent among men than women (56.8% vs. 46.6%; χ^2^ = 4.35, *p* = 0.037) (Table 2). In contrast, no sex-related differences were observed for subsequent injuries (χ^2^ = 1.27, *p* = 0.259), recurrent injuries (χ^2^ < 0.01, *p* = 0.998), or other subsequent-injury categories (χ^2^ = 1.18, *p* = 0.277).

Overall, subsequent injuries were reported by 34.9% of participants (n = 146). This category comprised both recurrent injuries affecting the same anatomical location and injury type, and additional injuries involving different locations or mechanisms. Consequently, a single participant could be classified simultaneously as having recurrent and other subsequent injuries during the same 12-month observation period. Accordingly, the “subsequent injury” category represents a broader grouping of repeated and multiple injury events occurring after an initial injury episode.

No statistically significant sex differences were observed for trunk injuries (χ^2^ = 0.05, *p* = 0.829), upper limb injuries (χ^2^ = 1.22, *p* = 0.269), lower limb injuries (χ^2^ = 3.12, *p* = 0.077), joint sprains (χ^2^ = 0.64, *p* = 0.422), bone fractures (χ^2^ < 0.01, *p* = 0.988), muscle strains (χ^2^ = 0.56, *p* = 0.452), or skin abrasions (χ^2^ = 0.22, *p* = 0.639). Nevertheless, lower limb injuries were the most frequently reported anatomical injury location in both sexes, particularly among men (49.2% vs. 40.6% among women).

No significant sex-related differences were observed for total physical activity (TPA) categories (χ^2^ = 2.56, *p* = 0.279). In contrast, significant differences between males and females were identified for training volume categories (χ^2^ = 11.22, *p* = 0.011), training age categories (χ^2^ = 11.87, *p* = 0.003), biomechanical–physiological discipline categories (χ^2^ = 21.05, *p* < 0.001), contact exposure categories (χ^2^ = 13.74, *p* < 0.001), and sport participation structure (χ^2^ = 13.40, *p* < 0.001). Males were more frequently involved in contact and team sports, whereas females were more commonly represented in non-contact and individual sport disciplines (Table 3).

### 3.2. Correlation Structure of Injury, Body Composition, and Training-Related Variables

Kendall’s Tau analysis revealed predominantly weak associations between body composition characteristics and injury outcomes, although several correlations reached statistical significance (Supplementary Table S1). Correlation coefficients were generally small (τ < 0.20), indicating limited pairwise relationships between individual variables. The relatively highest positive associations were observed for skeletal muscle-related indicators and injury outcomes, particularly between lower limb segmental muscle mass and injury occurrence (left lower limb: τ = 0.183, *p* < 0.001; right lower limb: τ = 0.182, *p* < 0.001). Similarly, skeletal muscle mass index (SMI) demonstrated its strongest association with upper limb injuries (τ = 0.192, *p* < 0.001), whereas total body water (TBW) showed the highest correlation with skin abrasions (τ = 0.187, *p* < 0.001).

Overall, SMI, TBW, and segmental muscle mass variables demonstrated the most consistent positive associations with injury-related outcomes, although the magnitude of these relationships remained weak. All observed associations remained small in magnitude despite statistical significance. In contrast, fat mass index (FMI), asymmetry indicators, and training-related variables demonstrated negligible or inconsistent correlations across the analyzed injury domains. Total physical activity and training volume were only weakly associated with injury outcomes.

### 3.3. Multidimensional Structure of Training and Demographic Characteristics

**Figure 3:**
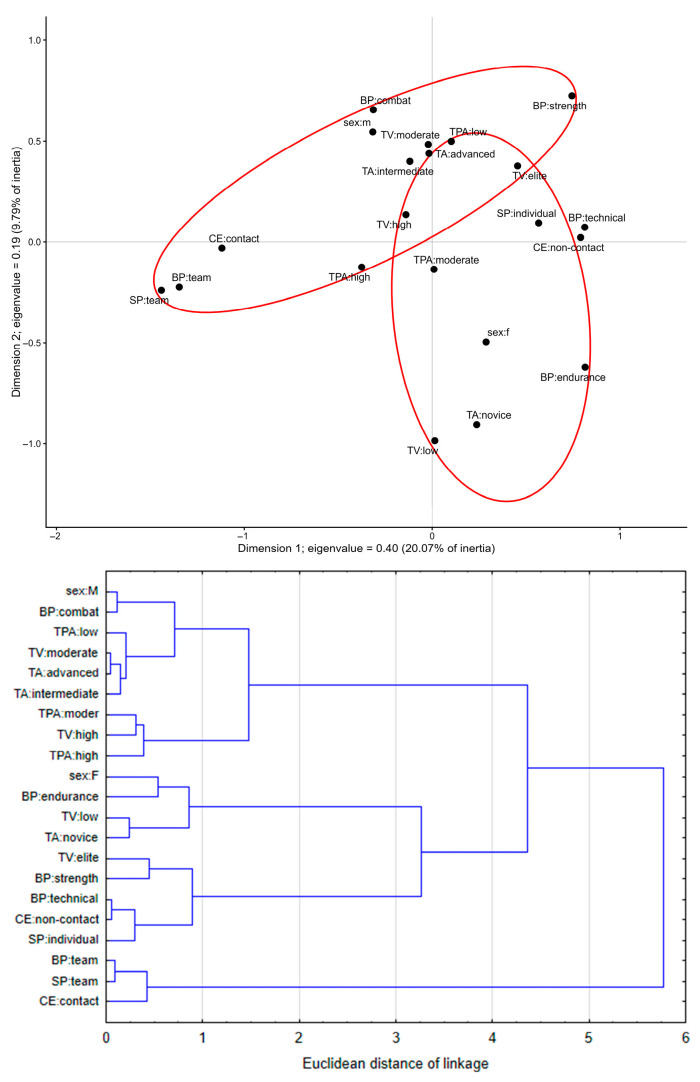
Multiple correspondence analysis (MCA) of training demographic characteristics and sport participation profiles. The first dimension explained 20.07% of inertia (eigenvalue = 0.40), whereas the second dimension accounted for 9.79% of inertia (eigenvalue = 0.19). Together, the first two dimensions explained 29.86% of the total inertia. Categories positioned closer in the multidimensional space demonstrated stronger co-occurrence patterns and greater multidimensional similarity. The horizontally oriented ellipse highlights the primary separation along Dimension 1, whereas the vertically oriented ellipse emphasizes differentiation along Dimension 2. Categories grouped within each ellipse represent profiles sharing similar positions on the corresponding MCA axis and therefore greater multidimensional similarity. Taxonomical dendrogram illustrates similarities and distances (strength) of linkage. Note: TPA—total physical activity category; TV—training volume category; TA—training age/training experience category; BP—biomechanical–physiological discipline category; CE—contact exposure category; SP—sport participation structure. BP categories included team, endurance, combat, strength, and technical disciplines. CE categories included contact and non-contact sports. SP categories included individual and team sports.

The MCA revealed a multidimensional organization of training demographic characteristics within the analyzed cohort (Figure 3). The first dimension explained 20.07% of inertia (eigenvalue = 0.40), whereas the second dimension accounted for 9.79% of inertia (eigenvalue = 0.19), resulting in a cumulative explained inertia of 29.86%.

Male participants were positioned closer to combat and strength-oriented disciplines, higher training exposure categories, contact sports, and team-based sport participation. In contrast, female participants were located closer to endurance disciplines, lower training volume, novice training experience, and non-contact sport categories. Categories associated with moderate and high total physical activity occupied more central positions within the MCA space, suggesting a weaker contribution to the observed multidimensional structure.

The first dimension primarily differentiated contact/team-oriented and strength combat sport profiles from endurance and lower-training-exposure profiles, whereas the second dimension appeared to reflect variation related to training advancement and training exposure. Categories located further from the centroid demonstrated greater discriminatory contribution and higher representation quality within the MCA solution.

The accompanying hierarchical clustering dendrogram was broadly consistent with the multidimensional structure observed in the MCA. Clustering patterns were apparent for contact and team sport participation, whereas endurance and non-contact categories formed a separate branch associated with lower training exposure and female sex. In contrast, strength and combat disciplines were positioned closer to the male sex and higher training exposure categories. The linkage distances suggested heterogeneous and partially differentiated sport training profiles within the analyzed population.

Overall, the MCA and hierarchical clustering analyses suggested partially differentiated sport training profiles characterized by recurring co-occurrence patterns among sex, training exposure, and sport participation structure. To further explore multidimensional similarity patterns among continuous body composition, training context, and injury-related variables, heatmap-based correlation and hierarchical clustering analyses were subsequently conducted.

### 3.4. Hierarchical Clustering and Heatmap Analysis of Morphological, Training, and Injury Variables

**Figure 4:**
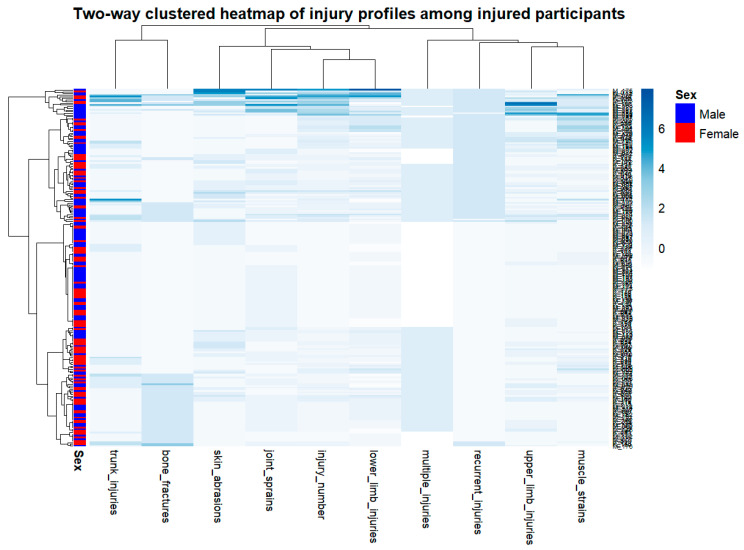
Heatmap-based hierarchical clustering of body composition, training, and injury profiles. The heatmap presents standardized values of body composition, segmental muscle mass, asymmetry, training-related variables, and injury outcomes across participants. Rows represent individual participants and columns represent analyzed variables. Hierarchical clustering was performed using Euclidean distances and Ward’s method. Sex was included as a row annotation to provide a biological demographic context for the observed clustering structure. Note: BH—body height; BW—body weight; BMI—body mass index; FMI—fat mass index; FFMI—fat-free mass index; SMI—skeletal muscle mass index; BM—bone mass; TBW—total body water; MM—muscle mass; RUL—right upper limb; LUL—left upper limb; RLL—right lower limb; LLL—left lower limb; ULA—upper limb asymmetry; LLA—lower limb asymmetry; TPA—total physical activity; TV—training volume; TA—training age/training experience; INJ—injury; SUB-INJ—subsequent injury; REC-INJ—recurrent injury; OS-INJ—other subsequent injury; TR-INJ—trunk injury; UL-INJ—upper limb injury; LL-INJ—lower limb injury; SPR—joint sprain; FRAC—bone fracture; STR—muscle strain; ABR—skin abrasion; BP—biomechanical–physiological discipline category; CE—contact exposure; SP—sport participation structure.

The two-way clustered heatmap suggested three exploratory participant-level injury profile patterns differing in injury burden and structural composition (Figure 4). Cluster 1 (n = 135) was characterized primarily by lower limb injuries and joint sprains, with limited recurrence and multisite involvement. Cluster 2 (n = 64) was characterized by relatively higher frequencies of muscle strains, recurrent injuries, and combined upper and lower limb involvement. Cluster 3 (n = 16) was characterized by a higher frequency of recurrent and multiple injuries, more extensive upper and lower limb involvement, frequent joint sprains and muscle strains, and elevated skin abrasions.

A progressive increase in the proportion of male participants was observed across clusters, rising from 48.9% in Cluster 1 to 68.7% in Cluster 3. Bone fractures appeared relatively isolated from the dominant recurrent multisite injury pattern and were not a prominent characteristic of the highest-burden cluster.

Additional exploratory analyses examined whether the identified injury profile patterns differed in body composition characteristics. Despite substantial differences in injury burden, recurrence, multisite involvement, and injury type composition, the clusters demonstrated relatively similar global body composition profiles, with only minor and inconsistent differences across adiposity, muscularity, hydration, and asymmetry indices.

These findings suggest that body composition characteristics alone may not adequately account for the observed variation among injury profile patterns. Therefore, the following section additionally incorporates training context variables, which may provide further insight into the multidimensional context associated with injury occurrence and injury profile variability.

### 3.5. Concordance Between Body Composition Phenotypes and Injury Patterns: Tanglegram-Based Clustering Analysis

#### 3.5.1. Global BC vs. Injuries

**Figure 5:**
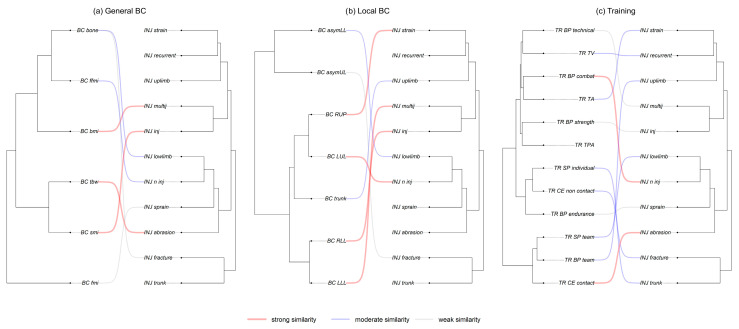
Topology-based cross-domain matching between body composition, training context, and injury-related phenotypes. (**a**) Tanglegram illustrating optimal structural matching between global body composition (BC) variables and injury-related variables. (**b**) Tanglegram illustrating optimal structural matching between local/segmental body composition variables and injury-related variables. (**c**) Tanglegram illustrating optimal structural matching between training context and injury-related variables. Cross-domain correspondences were identified using the Hungarian algorithm based on Euclidean distances derived from standardized multidimensional variable profiles. Colored links represent relative similarity strength between paired variables (red = strong similarity; blue = moderate similarity; gray = weak similarity). Colored links represent relative similarity strength between paired variables (red = strong similarity; blue = moderate similarity; gray = weak similarity). For visualization purposes, only strong and moderate similarity links are displayed, whereas weaker links were omitted to improve figure readability. All matches were generated algorithmically using the Hungarian optimization procedure. For training context analyses, categorical variables were transformed into dummy-coded binary indicators prior to clustering procedures. For training context analyses, categorical variables were transformed into dummy-coded binary indicators prior to clustering procedures. Note: BC_bone—bone mass; BC_FFMI—fat-free mass index; BC_TBW—total body water; BC_SMI—skeletal muscle index; BC_FMI—fat mass index; BC_BMI—body mass index; INJ_strain—muscle strain; INJ_recurrent—recurrent injuries; INJ_uplimb—upper limb injuries; INJ_multij—multi-site injuries; INJ_inj—any injury occurrence; INJ_lowlimb—lower limb injuries; INJ_n_inj—number of injuries; INJ_sprain—joint sprain; INJ_abrasion—skin abrasion; INJ_fracture—bone fracture; INJ_trunk—trunk injuries; BC_asym_LL—lower limb muscular asymmetry; BC_asym_UL—upper limb muscular asymmetry; BC_RUL—right upper limb muscle mass; BC_LUL—left upper limb muscle mass; BC_trunk—trunk muscle mass; BC_RLL—right lower limb muscle mass; BC_LLL—left lower limb muscle mass; TR_BP_technical—technical sports profile; TR_TV—training volume; TR_BP_combat—combat sports profile; TR_TA—training age; TR_BP_strength—strength sports profile; TR_TPA—total physical activity; TR_SP_individual—individual sport participation; TR_CE_non_contact—non-contact sport exposure; TR_BP_endurance—endurance sports profile; TR_SP_team—team sport participation; TR_BP_team—team sports profile; TR_CE_contact—contact sport exposure.

The global BC tanglegram suggested partial similarity between body composition and injury-related clustering structures (Figure 5a). Lean mass-related variables, particularly FFMI and bone mass, were paired most closely with upper limb injuries and muscle-strain patterns within the matching procedure, whereas BMI and TBW (%) were paired more closely with multisite injuries, overall injury occurrence, and lower limb injuries. In contrast, FMI demonstrated comparatively weak and less differentiated similarity patterns across injury-related variables. The injury dendrogram additionally contained several subclusters, including (a) recurrent injuries with muscle strains, (b) lower limb injuries with overall injury occurrence, and (c) trunk injuries with fractures.

Cophenetic correlation coefficients indicated acceptable structural representation for the global BC tree (CCC = 0.815) and moderate representation for the injury tree (CCC = 0.644). Link-level cophenetic concordance analyses demonstrated weak inverse cross-domain agreement between global BC and injury patterns (Spearman = −0.264; Kendall = −0.233), indicating limited overall topological similarity despite several prominent pairwise matches identified by the algorithmic matching procedure.

#### 3.5.2. Local BC vs. Injuries

The local BC tanglegram suggested more differentiated similarity patterns between segmental body composition variables and injury-related profiles than were observed in the global BC analysis (Figure 5b). Upper limb variables were paired with upper limb injury profiles within the optimal structure-matching solution, although these links should be interpreted as topological correspondences rather than direct biological relationships. Trunk-related variables demonstrated intermediate similarity to general injury patterns. Compared with the global BC analysis, the local BC structure appeared more anatomically differentiated, whereas asymmetry variables demonstrated weaker and less specific similarity patterns across injury-related variables.

Cophenetic correlation coefficients indicated very high structural fidelity of the local BC dendrogram (CCC = 0.965), whereas the injury tree again demonstrated moderate representation (CCC = 0.644). Link-level cophenetic concordance analyses demonstrated moderate positive cross-domain agreement between local BC and injury profiles (Spearman = 0.549; Kendall = 0.484), indicating greater topological similarity than observed for the global BC analysis.

#### 3.5.3. Training Context vs. Injuries

The training context tanglegram suggested diffuse similarity patterns between training-related characteristics and injury profiles (Figure 5c). Continuous indicators of cumulative training exposure, including total physical activity (TPA), training volume (TV), and training age (TA), were paired most closely with recurrent injuries, lower limb injuries, and overall injury occurrence within the matching procedure. In contrast, dummy-coded categorical descriptors related to sport profile and body-contact exposure formed more compact structures within the training dendrogram. Although several pairwise matches were highlighted in the visualization, the overall structure appeared diffuse and less anatomically differentiated than the body composition-based tanglegrams.

Cophenetic correlation coefficients demonstrated moderate structural representation for both the training tree (CCC = 0.672) and injury tree (CCC = 0.644). Link-level cophenetic concordance analyses demonstrated minimal topological agreement between training context and injury profiles (Spearman = 0.030; Kendall = 0.022), indicating very limited overall cross-domain similarity despite the presence of several pairwise matches identified by the matching procedure.

Overall, the topology-based matching analyses suggested partial similarity patterns between body composition, training context, and injury-related clustering structures. Among the analyzed domains, local body composition variables demonstrated the greatest degree of topological similarity to injury-related clustering structures.

Because tanglegrams evaluate similarities in hierarchical organization rather than statistical associations, the identified pairings should be interpreted as exploratory topological correspondences and hypothesis-generating observations requiring independent validation.

### 3.6. Distance-Based Redundancy Analysis (dbRDA): Multivariate Associations Between Body Composition, Training Context, and Injury Patterns

Distance-based redundancy analysis was used to evaluate whether multidimensional injury phenotypes were associated with body composition and training context characteristics. The body composition model demonstrated a statistically significant but weak association with injury phenotypes (R^2^ = 0.057, adjusted R^2^ = 0.027, F = 1.89, *p* = 0.001), indicating that body composition variables explained only a small proportion of injury-related variability (Table 4). In contrast, the training context model was not statistically significant (R^2^ = 0.024, adjusted R^2^ = 0.002, F = 1.11, *p* = 0.304), suggesting that training-related characteristics accounted for very little injury-related variability. The combined model reached statistical significance (R^2^ = 0.073, adjusted R^2^ = 0.022, F = 1.42, *p* = 0.023), although the explained variance remained low, indicating limited explanatory capacity of the analyzed predictors (Table 4).

**Figure 6:**
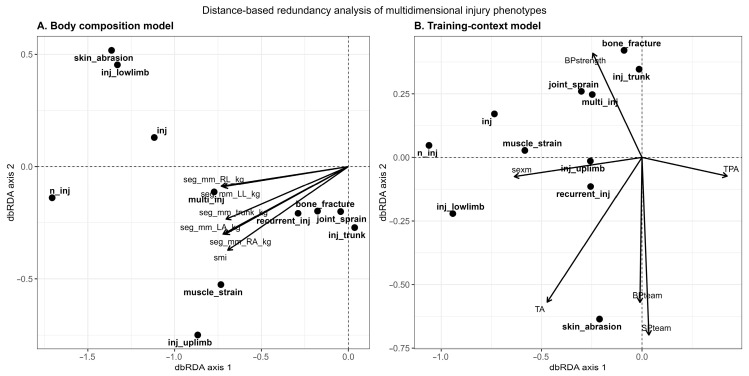
Distance-based redundancy analysis (dbRDA) ordination of multidimensional injury phenotypes constrained separately by body composition variables (**A**) and training context variables (**B**). Only the strongest explanatory vectors are displayed to improve ordination readability and reduce graphical overplotting. The body composition model showed a statistically significant but weak multivariate association with injury phenotypes, whereas the training context model was not significant. Overall, the ordination indicated diffuse and weakly structured injury-related gradients, suggesting limited constrained variance explained by the analyzed predictors. Note: BC_bone—bone mass; BC_FFMI—fat-free mass index; BC_TBW—total body water; BC_SMI—skeletal muscle index; BC_FMI—fat mass index; BC_BMI—body mass index; INJ_strain—muscle strain; INJ_recurrent—recurrent injuries; INJ_uplimb—upper limb injuries; INJ_multij—multi-site injuries; INJ_inj—any injury occurrence; INJ_lowlimb—lower limb injuries; INJ_n_inj—number of injuries; INJ_sprain—joint sprain; INJ_abrasion—skin abrasion; INJ_fracture—bone fracture; INJ_trunk—trunk injuries; BC_asym_LL—lower limb muscular asymmetry; BC_asym_UL—upper limb muscular asymmetry; BC_RUL—right upper limb muscle mass; BC_LUL—left upper limb muscle mass; BC_trunk—trunk muscle mass; BC_RLL—right lower limb muscle mass; BC_LLL—left lower limb muscle mass; TR_BP_technical—technical sports profile; TR_TV—training volume; TR_BP_combat—combat sports profile; TR_TA—training age; TR_BP_strength—strength sports profile; TR_TPA—total physical activity; TR_SP_individual—individual sport participation; TR_CE_non_contact—non-contact sport exposure; TR_BP_endurance—endurance sports profile; TR_SP_team—team sport participation; TR_BP_team—team sports profile; TR_CE_contact—contact sport exposure.

The ordination demonstrated weak and diffuse injury-related gradients, with no clear separation of injury phenotypes (Figure 6). These results suggest that injury patterns in the studied population were only weakly structured by isolated body composition and training context characteristics, supporting the interpretation of injury occurrence as a heterogeneous and multifactorial phenomenon. However, the low adjusted R^2^ values indicate that the analyzed variables accounted for only a small proportion of injury-related variability. Therefore, the observed associations should be interpreted as exploratory structural relationships rather than evidence of substantial explanatory or predictive value.

## 4. Discussion

The present study demonstrated weak but interpretable multidimensional relationships between injury occurrence, body composition, and training-related characteristics in physically active young adults. Segmental body composition variables appeared to show somewhat stronger associations with injury localization patterns than global indices, although the magnitude of these relationships remained limited. Nevertheless, the overall cross-domain structure remained diffuse and weakly separated, supporting the interpretation of injury occurrence as a predominantly multifactorial and weakly organized phenomenon within the studied population. This interpretation was further supported by dbRDA models, indicating that body composition and training context characteristics explained only a limited proportion of overall injury-related variability. Accordingly, the present findings should be interpreted cautiously, as statistically significant associations did not necessarily translate into strong explanatory capacity at the population level.

### 4.1. Body Composition as a Partial but Non-Dominant Component of Injury-Related Variability

Selected body composition variables demonstrated weak but significant associations with injury-related outcomes, particularly with lower limb and recurrent injuries. The observed relationships suggest that morphological characteristics may be associated with injury occurrence patterns; however, their explanatory role appears limited when considered in isolation. This interpretation is consistent with contemporary multifactorial and dynamic models of sports injury etiology, which emphasize that injuries emerge from the interaction of multiple intrinsic and extrinsic factors rather than from single, isolated injury-associated characteristics. Previous studies have proposed that body composition characteristics may be associated with injury occurrence through mechanisms related to mechanical loading, movement efficiency, tissue stress, and neuromuscular imbalance. However, such mechanisms were not directly assessed in the present study. For example, Martins et al. [30] reported that injury status in professional soccer players was associated with seasonal variations in body fat percentage, skeletal muscle mass, and total body water, suggesting that body composition characteristics may reflect both injury occurrence patterns and adaptive physiological responses to training and competition. Similarly, Ali et al. [31] observed that higher body fat mass and body fat percentage were associated with greater injury incidence, although the predictive effects remained relatively weak. A recent systematic review by Melloni et al. [26] further concluded that body composition indicators such as BMI and body mass may contribute to injury occurrence, particularly ankle sprains, but emphasized the inconsistency and limited quality of available evidence regarding more specific composition variables.

In the present study, segmental and asymmetry-related indicators appeared more informative than global anthropometric indices alone, particularly in relation to lower limb and recurrent injuries. This observation may be consistent with previous reports linking localized biomechanical imbalance and kinetic chain disturbances with injury occurrence; however, the present data do not permit conclusions regarding underlying biomechanical mechanisms [32]. Lévesque et al. [33] showed that side-to-side asymmetry in lumbar multifidus muscle cross-sectional area was associated with lower limb injury history, supporting the relevance of localized muscular imbalance in injury-related processes. Nevertheless, despite these partial associations, the multidimensional analyses performed in the present study did not reveal strongly separated injury profiles based solely on body composition characteristics. This may indicate that body composition represents only one component of a broader multidimensional injury-related system involving training exposure, sport-specific demands, functional characteristics, and other unmeasured behavioral or biomechanical factors.

### 4.2. Segmental Asymmetry and Localized Injury Patterns

The observed associations between asymmetry-related variables and selected injury outcomes are consistent with the possibility that localized biomechanical imbalance may be associated with injury occurrence patterns, particularly in relation to lower limb and recurrent injuries. However, the present findings should be interpreted cautiously, as the observed relationships appeared partial rather than deterministic. Previous literature has produced inconsistent results regarding the role of asymmetry in injury occurrence. A systematic review by Helme et al. [34] concluded that although asymmetry may contribute to injury occurrence, the overall quality and consistency of evidence remain moderate to low. Similarly, Guan et al. [35] emphasized that substantial methodological heterogeneity limits definitive conclusions regarding asymmetry-based injury prediction.

Nevertheless, several studies support the potential relevance of localized asymmetry and functional imbalance in physically active populations. Koźlenia et al. [36] demonstrated that lower limb skeletal muscle mass asymmetry and force asymmetry were associated with elevated injury occurrence, although the relevant predictors differed between sexes. Consistently, Eagle et al. [37] reported that asymmetry alone had limited predictive value but became relevant when analyzed together with body composition characteristics such as body mass and BMI. Additionally, Hart et al. [38] suggested that chronic sport-specific loading may progressively contribute to the development of musculoskeletal asymmetries. Collectively, these findings suggest that asymmetry represents one component of a broader multidimensional injury-related framework rather than an isolated contributing factor.

### 4.3. Training Demographic Context as an “Injury Ecosystem”

The multidimensional analyses performed in the present study suggest that injury-related variability cannot be fully understood through isolated morphological characteristics alone, but should also be considered within a broader training demographic and sport-specific context. The MCA results demonstrated partially differentiated participant profiles associated with sex, sport type, and training characteristics, indicating that injury-related patterns may be associated with combinations of multiple contextual factors. This interpretation is consistent with contemporary ecological and systems-based approaches to sports injury etiology, which emphasize that injuries arise through dynamic interactions among biological, behavioral, environmental, and sport-specific components rather than through single isolated mechanisms. Hulme and Finch [39] argued that traditional reductionist approaches may insufficiently capture the complexity of injury processes and proposed a systems thinking framework integrating multidimensional interactions across athlete environments. Similarly, Jacobsson et al. [40] highlighted that injury development reflects interactions across multiple ecological levels, including athlete behavior, training context, and environmental conditions.

The present findings suggest that sport-specific exposure and training characteristics contribute to differences in participant profiles; however, their independent association with overall injury-related variability appeared limited. Therefore, training context characteristics may represent only one component of the broader behavioral and sport participation context associated with injury occurrence. Previous research has shown that both excessive and insufficient training exposure may be associated with greater injury occurrence depending on the broader adaptive context. Correspondingly, Johnston et al. [18] demonstrated that injury occurrence may depend on interactions between training load characteristics and baseline athlete-related factors. Additionally, Leventer et al. [41] suggested that contact injuries arise through dynamic performer–environment interactions rather than isolated mechanical events. Collectively, these observations support the interpretation that the multidimensional patterns observed in the present study are consistent with the complex and context-dependent nature of injury-related variability in physically active young adults.

Nevertheless, the dbRDA model based on training context variables was not statistically significant, indicating that these characteristics contributed primarily to descriptive profile differentiation rather than to overall injury-related variability. Consequently, interpretations regarding training context influences should be regarded as exploratory and hypothesis-generating.

### 4.4. Heterogeneity of Injury and Training Profiles in Physically Active Young Adults

The multidimensional and overlapping participant profiles observed in the present study further support the concept that injury occurrence reflects complex and heterogeneous interactions among biological, functional, psychological, and training-related factors. Although some injury profile patterns appeared partially differentiated, substantial overlap among clusters suggests that physically active individuals may follow multiple pathways associated with injury occurrence. This interpretation is consistent with biopsychosocial and multidimensional models of sports injury etiology. Rosen et al. [42] demonstrated that injury occurrence may result from interactions among training load, sleep, psychological characteristics, and behavioral factors rather than from isolated predictors alone. Similarly, Taimela et al. [43] proposed that injury occurrence patterns reflect a complex network of intrinsic factors, including biomechanics, fitness, psychological characteristics, previous injury history, and training exposure.

The injury profile patterns identified in the present study may additionally support the relevance of person-centered and profile-based approaches in sports injury research. Levin et al. [44] reported that distinct combinations of psychological and physical characteristics were associated with different injury occurrence patterns, emphasizing the importance of multidimensional profiling. Similarly, Martin et al. [45] suggested that overuse injuries may arise through interacting psychological configurations rather than from isolated injury-related factors. Additionally, Maruszyńska-Małachowska and Kamiński [46] demonstrated that injury profiles differ substantially according to sport-related exposure and injury location. 

Altogether, these findings suggest that the heterogeneous and overlapping patterns observed in the present analyses are consistent with the inherently multidimensional and context-dependent nature of injury-related variability in physically active populations. Nevertheless, the identified profile patterns should be regarded as exploratory and hypothesis-generating rather than as definitive injury classifications.

### 4.5. Injury Burden Beyond Isolated Injury Events

The present findings may also support the importance of considering injury burden and multidimensional injury profile rather than focusing exclusively on isolated injury events. Although the identified clusters were only partially differentiated, recurrent injuries, multi-site injuries, and greater injury accumulation tended to co-occur within specific participant profiles. This observation is consistent with contemporary approaches emphasizing that injury burden reflects not only injury incidence but also the cumulative impact of repeated and time-loss-related injuries on athlete functioning and availability. Bahr et al. [47] argued that injury incidence alone may provide an incomplete representation of athlete risk and highlighted the importance of considering injury severity and cumulative burden when evaluating injury-related outcomes. The profile-based patterns observed in the present analyses may additionally support the usefulness of multidimensional athlete phenotyping approaches in injury research. Pérez-Murillo et al. [48] identified distinct preseason body composition phenotypes associated with different levels of in-season injury burden, suggesting that multidimensional profiling may help identify athletes potentially predisposed to greater injury accumulation. Although the present study did not reveal fully discrete injury categories, the patterns observed through clustering and heatmap analyses are consistent with the possibility that injury burden reflects interactions among biological, training-related, and other contextual characteristics rather than isolated determinants. In a broader context, Liu et al. [49] demonstrated the growing scientific interest in injury burden as a multidimensional concept integrating epidemiological, clinical, and risk-factor perspectives. Taken together, these observations align with the interpretation that multidimensional and profile-oriented analytical frameworks may provide additional insight into injury-related variability beyond traditional single-outcome approaches.

### 4.6. Structural Concordance Between Body Composition, Training Context, and Injury Domains

The partial similarity patterns observed between domains should be interpreted as exploratory topological correspondences rather than evidence of direct biological linkages among body composition, training context, and injury-related variables. The principal value of the tanglegram framework lies in its ability to compare multidimensional organizational patterns across domains rather than focusing exclusively on within-domain clustering or ordination results. Rather than reflecting simple linear relationships, the observed similarity patterns may be consistent with the multidimensional organization of injury-related variability involving biological, functional, and contextual domains. This interpretation is consistent with contemporary complex systems approaches in sports medicine. Thuany et al. [50] demonstrated that training-related characteristics may exert stronger influences on injuries than isolated anthropometric variables when analyzed within network-based models. Similarly, Pol et al. [51] proposed that injuries arise through nonlinear interactions and the accumulation of constraints operating across multiple organizational levels.

The present findings may also support the relevance of multidimensional and interdisciplinary approaches to injury prevention and management. Tee et al. [52] demonstrated that injury reduction strategies may benefit from integrating multiple professional domains within iterative prevention frameworks. Likewise, Hulme et al. [53] emphasized that systems-oriented approaches may improve understanding of injury occurrence and management by accounting for interactions among athletes, training environments, and organizational contexts.

Taken together, these observations suggest that the multidimensional analytical framework applied in the present study provides a useful exploratory perspective for investigating complex injury-related systems in physically active populations. Nevertheless, the topological correspondences identified in the present analyses should be regarded as exploratory and hypothesis-generating and require validation in independent datasets.

### 4.7. Methodological Novelty and Multidimensional Analytical Framework

The multidimensional analytical framework applied in the present study may represent a methodological contribution to contemporary sports injury research. By integrating MCA, hierarchical clustering, heatmaps, tanglegram-based topology matching, and dbRDA, the study moved beyond traditional single-variable approaches and enabled exploration of multidimensional patterns linking body composition, training context, and injury-related variability. Recent studies increasingly emphasize the value of multivariate and unsupervised analytical frameworks for athlete monitoring and injury-related profiling. Luca et al. [54] demonstrated that unsupervised multidimensional approaches may identify latent physiological and injury-related phenotypes not readily detectable using conventional univariate methods. Similarly, Ma [55] highlighted the utility of multidimensional clustering and data-mining approaches for characterizing heterogeneous injury risk profiles across athletes.

The growing integration of machine-learning techniques and multidomain analytical frameworks in sports science further supports the relevance of exploratory multidimensional approaches. Jianjun et al. [56] demonstrated that combining physiological, biomechanical, psychological, and contextual variables may improve characterization of athlete-related variability compared with traditional models. Similarly, Iduh et al. [57] emphasized the importance of integrating demographic, training, and performance characteristics into individualized injury risk profiling systems.

Although the present study was exploratory rather than predictive, the applied analytical framework may provide a useful basis for future hypothesis-generating investigations and for the development of multidimensional athlete monitoring strategies. Future studies should evaluate the stability and reproducibility of the identified profile patterns using independent samples and resampling-based validation procedures.

### 4.8. Interpretation of Relatively Weak Effect Sizes

Given the weak magnitude of the observed associations and the retrospective cross-sectional design, the present findings should not be interpreted as evidence of causal or mechanistic relationships between body composition characteristics and injury occurrence. 

The predominantly weak associations observed in the present study are consistent with the complex and multifactorial nature of sports injuries. Previous methodological and theoretical frameworks have emphasized that injury occurrence rarely results from isolated determinants and instead reflects interactions among multiple dynamic and time-varying factors. Bahr and Holme [58] highlighted that sports injuries emerge from complex interactions between numerous injury-related factors and that small-to-moderate associations are difficult to detect without large samples and multivariate approaches. Similarly, Nielsen et al. [59] emphasized the dynamic and fluctuating nature of injury etiology, particularly in relation to changing training exposures over time.

The present findings may additionally support contemporary complex systems perspectives in sports injury research. Hulme et al. [60] argued that traditional linear models may insufficiently capture the interconnected and feedback-driven nature of injury development. Likewise, Pol et al. [52] proposed that injuries emerge through nonlinear interactions and the accumulation of constraints across multiple organizational levels. Furthermore, psychosocial and behavioral factors not directly assessed in the present study may also contribute substantially to injury-related variability. Ivarsson et al. [61] demonstrated that stress-related and psychosocial variables may meaningfully influence injury occurrence. Taken together, these observations may help contextualize why the associations observed in the present study remained relatively weak despite the use of multidimensional analytical approaches. This interpretation is further supported by the low adjusted R^2^ values observed in the dbRDA analyses, indicating that a large proportion of injury-related variability remained unexplained by the variables included in the present models.

### 4.9. Practical Implications, Strengths, and Limitations

The present findings suggest that isolated anthropometric screening may provide only limited insight into multidimensional injury-related variability. Segmental body composition and asymmetry-related indicators appeared to demonstrate somewhat stronger associations with selected injury-related patterns than global indices alone; however, the observed relationships remained weak. Therefore, multidimensional profiling approaches may represent a useful exploratory framework for future injury-related investigations. Nevertheless, prospective and longitudinal studies are required before practical monitoring, screening, or prevention applications can be recommended.

The strengths of the present study include the multidimensional analytical framework integrating correlation analyses, multiple correspondence analysis (MCA), hierarchical clustering, heatmap-based profiling, topology-based cross-domain matching using tanglegrams, and distance-based redundancy analysis (dbRDA). This approach enabled simultaneous evaluation of global and local body composition characteristics, training context variables, and multidimensional injury profile patterns within a relatively large cohort of physically active young adults. The inclusion of both continuous and categorical sport training descriptors additionally allowed exploration of injury-related patterns across morphological, behavioral, and sport participation domains.

Several limitations should also be acknowledged. First, the cross-sectional design precludes causal inference regarding the observed associations between body composition, training exposure, and injury occurrence. Second, injury history and physical activity measures were based on self-reported data and may therefore be affected by recall bias, misclassification bias, and reporting inaccuracies. Participants may have forgotten minor injuries or inaccurately reported injury type, anatomical location, or recurrence status. Such inaccuracies may have introduced additional variability into the dataset, potentially influencing the observed clustering patterns and the topology-based matching results obtained from the tanglegram analyses. Consequently, some multidimensional patterns identified in the present study should be interpreted with appropriate caution. Third, the injury variables demonstrated relatively sparse and heterogeneous distributions, which may have reduced the robustness of the multidimensional clustering patterns. Fourth, despite several statistically significant multidimensional associations, the explained variance in dbRDA models and the cophenetic concordance between cross-domain structures remained relatively modest, indicating that substantial injury-related variability was likely attributable to unmeasured biomechanical, psychological, technical, environmental, and sport-specific factors. An additional limitation is that the injury history questionnaire included injuries related to sport participation, recreational activities, and daily life events. Consequently, not all recorded injuries were necessarily attributable to training or sport exposure. This may have reduced the strength of associations with training context variables and contributed to the diffuse multidimensional structures observed in the study. Furthermore, the stability and reproducibility of the identified injury profile patterns were not formally evaluated using resampling or bootstrapping procedures. Therefore, the observed profile patterns should be interpreted as exploratory and hypothesis-generating and require confirmation in independent datasets. Finally, the study population consisted exclusively of physically active university students, which may limit generalizability to elite athletes, sedentary populations, or clinical cohorts.

### 4.10. Future Directions

Future studies should incorporate prospective longitudinal injury surveillance designs to better characterize temporal relationships between morphological characteristics, training exposure, and injury occurrence. Prospective validation studies will be necessary to determine whether the multidimensional profile patterns identified in the present study are reproducible across independent populations and whether they possess practical relevance for injury-related research.

Future investigations should additionally evaluate the stability and reproducibility of clustering results using resampling- and bootstrapping-based validation procedures. The inclusion of biomechanical, neuromuscular, psychological, recovery-related, and sport-specific load-monitoring variables may further improve multidimensional characterization of injury-related patterns. Additionally, future research may benefit from integrating machine-learning, network-based, and trajectory modeling approaches to identify more complex and dynamic interaction patterns.

Particular attention should also be directed toward individualized and sport-specific profiling, as the present findings suggest that injury occurrence reflects diffuse and heterogeneous multifactorial processes rather than clearly separated body composition or training-related profile patterns.

## 5. Conclusions

The findings suggest that body composition characteristics were associated with multidimensional injury-related patterns, whereas training context variables demonstrated limited independent explanatory value in this cohort. Among the analyzed domains, local and segmental body composition variables demonstrated the greatest degree of topological similarity to injury-related clustering structures.

Multidimensional exploratory approaches, including MCA, hierarchical clustering, topology-based cross-domain matching using tanglegrams, and dbRDA, revealed diffuse and overlapping injury-related patterns rather than clearly distinguishable injury profile categories. Although several statistically significant associations were identified, the overall explained variance and cross-domain cophenetic concordance remained relatively modest. Consequently, the findings should be viewed primarily as exploratory observations describing multidimensional organizational patterns rather than as definitive explanations or robust classifications of injury occurrence.

These findings are consistent with the interpretation of injury occurrence as a heterogeneous and multifactorial phenomenon; however, the weak associations and low explained variance indicate that most injury-related variability remains unexplained by the variables included in the present study. The study additionally demonstrates the utility of multidimensional exploratory frameworks for investigating complex injury-related systems beyond conventional single-factor approaches.

Injury occurrence in physically active young adults appears to reflect diffuse and weakly organized multidimensional interactions rather than isolated morphological determinants. These observations should be regarded as exploratory and hypothesis-generating and require confirmation in independent datasets before broader interpretation or practical application.

## Figures and Tables

**Table 1:** General characteristics of the study participants (N = 418).

Variable			Males n = 199			Females n = 219			
	Mean	95%CI Lower	95%CI Upper	SD	Mean	95%CI Lower	95%CI Upper	SD	*p*
Age [y]	20.73	20.61	20.85	0.85	20.56	20.46	20.65	0.74	**0.025**
Body height [cm]	182.19	181.20	183.18	7.10	168.17	167.37	168.97	6.01	**<0.001**
Body weight [kg]	79.63	78.25	81.01	9.87	60.86	59.65	62.06	9.05	**<0.001**
BMI [kg/m^2^]	23.97	23.62	24.32	2.48	21.49	21.12	21.85	2.71	**<0.001**
FMI [kg/m^2^]	3.92	3.72	4.12	1.40	5.09	4.85	5.33	1.82	**<0.001**
FFMI [kg/m^2^]	19.95	19.70	20.19	1.74	16.37	16.17	16.57	1.49	**<0.001**
SMI [kg/m^2^]	17.02	16.60	17.43	2.96	12.74	12.28	13.21	3.48	**<0.001**
BM [kg]	3.29	3.25	3.34	0.34	2.35	2.32	2.38	0.23	**<0.001**
TBW [%]	54.71	53.61	55.82	7.91	45.30	43.76	46.83	11.50	**<0.001**
Trunk muscle mass [kg]	30.47	29.66	31.29	5.81	20.96	20.16	21.76	6.00	**<0.001**
Right upper limb muscle mass [kg]	3.31	3.22	3.40	0.66	1.86	1.82	1.91	0.32	**<0.001**
Left upper limb muscle mass [kg]	3.32	3.22	3.42	0.70	1.84	1.80	1.89	0.33	**<0.001**
Right lower limb muscle mass [kg]	9.93	9.68	10.18	1.78	6.65	6.45	6.85	1.51	**<0.001**
Left lower limb muscle mass [kg]	9.62	9.37	9.86	1.73	6.49	6.30	6.69	1.47	**<0.001**
Upper limbs asymmetry [%]	2.10	1.85	2.34	1.74	2.49	2.14	2.83	2.59	**<0.001**
Lower limb asymmetry [%]	3.32	3.11	3.53	1.49	2.42	2.23	2.60	1.40	**<0.001**
TPA [MET/min/week]	3608.2	3418.5	3797.9	1356.9	3019.8	2886.5	3153.1	1000.9	**<0.001**
TV [h·week^−1^]	6.14	5.60	6.68	3.88	5.66	5.15	6.17	3.82	0.200
TA [years]	3.61	3.43	3.79	1.29	3.11	2.92	3.31	1.47	**<0.001**

Abbreviations: BMI—body mass index; FMI—fat mass index; FFMI—free fat mass index, SMI—skeletal muscle mass index; BM—bone mass, TBW—total body water, TPA—total physical activity calculated based on the International Physical Activity Questionnaire (IPAQ); TV—training weekly volume (expressed in hours per week); TA—training age (experience expressed in years); SD—standard deviation; CI—confidence interval. Statistically significant values are highlighted in bold.

**Table 2:** Frequency distributions of injury categories by sex (males: n = 199; females: n = 219).

Injury Category	Sex	0 (n, %)	1 (n, %)	χ^2^	*p*
Any injury	males	86 (43.2%)	113 (56.8%)	4.35	0.037
	females	117 (53.4%)	102 (46.6%)		
Subsequent injury	males	124 (62.3%)	75 (37.7%)	1.27	0.259
	females	148 (67.6%)	71 (32.4%)		
Recurrent injury	males	153 (76.9%)	46 (23.1%)	0.00	0.998
	females	184 (84.0%)	35 (16.0%)		
Other subsequent injury	males	131 (65.8%)	68 (34.2%)	1.18	0.277
	females	155 (70.8%)	64 (29.2%)		
Trunk injury	males	174 (87.4%)	25 (12.6%)	0.05	0.829
	females	193 (88.1%)	26 (11.9%)		
Upper limb injury	males	153 (76.9%)	46 (23.1%)	1.22	0.269
	females	178 (81.3%)	41 (18.7%)		
Lower limb injury	males	101 (50.8%)	98 (49.2%)	3.12	0.077
	females	130 (59.4%)	89 (40.6%)		
Joint sprain	males	128 (64.3%)	71 (35.7%)	0.64	0.422
	females	149 (68.0%)	70 (32.0%)		
Bone fracture	males	168 (84.4%)	31 (15.6%)	0.00	0.988
	females	185 (84.5%)	34 (15.5%)		
Muscle strain	males	153 (76.9%)	46 (23.1%)	0.56	0.452
	females	175 (79.9%)	44 (20.1%)		
Skin abrasion	males	160 (80.4%)	39 (19.6%)	0.22	0.639
	females	180 (82.2%)	39 (17.8%)		

Note: “1” indicates the presence of at least one injury within a given category, whereas “0” indicates its absence. Percentages are presented within sex-specific groups. χ^2^ values were calculated using Pearson’s chi-square test.

**Table 3:** Frequency distributions of physical activity, training, and sport participation characteristics according to sex (males: n = 199; females: n = 219).

Training Characteristic	Sex	Category 1 n (%)	Category 2 n (%)	Category 3 n (%)	Category 4 n (%)	Category 5 n (%)	χ^2^	*p*
TPA	males	High: 17 (8.5%)	Moderate: 134 (67.3%)	Low: 48 (24.1%)	—	—	2.56	0.279
	females	High: 15 (6.8%)	Moderate: 163 (74.4%)	Low: 41 (18.7%)	—	—		
TV	males	Elite: 17 (8.5%)	High: 31 (15.6%)	Moderate: 107 (53.8%)	Low: 44 (22.1%)	—	11.22	0.011
	females	Elite: 11 (5.0%)	High: 43 (19.6%)	Moderate: 91 (41.6%)	Low: 74 (33.8%)	—		
TA	males	Advanced: 16 (8.0%)	Intermediate: 137 (68.8%)	Novice: 46 (23.1%)	—	—	11.87	0.003
	females	Advanced: 20 (9.1%)	Intermediate: 116 (53.0%)	Novice: 83 (37.9%)	—	—		
BP	males	Team: 83 (41.7%)	Endurance: 33 (16.6%)	Combat: 18 (9.0%)	Strength: 41 (20.6%)	Technical: 24 (12.1%)	21.05	<0.001
	females	Team: 52 (23.7%)	Endurance: 62 (28.3%)	Combat: 20 (9.1%)	Strength: 41 (18.7%)	Technical: 44 (20.1%)		
CE	males	Non-contact: 98 (49.2%)	Contact: 101 (50.8%)	—	—	—	13.74	<0.001
	females	Non-contact: 147 (67.1%)	Contact: 72 (32.9%)	—	—	—		
SP	males	Individual: 126 (63.3%)	Team: 73 (36.7%)	—	—	—	13.40	<0.001
	females	Individual: 174 (79.5%)	Team: 45 (20.5%)	—	—	—		

Note: TPA—total physical activity; TV—training volume; TA—training age (training experience); BP—biomechanical–physiological discipline category; CE—contact exposure category; SP—sport participation structure. Percentages are presented within sex-specific groups. χ^2^ values were calculated using Pearson’s chi-square test.

**Table 4:** Summary of distance-based redundancy analysis models explaining multidimensional injury phenotypes.

Model	R^2^	adj R^2^	F	*p*	Interpretation
Body composition	0.057	0.027	1.89	0.001	Significant but weak association with limited explanatory capacity
Training context	0.024	0.002	1.11	0.304	No significant global association between training context variables and injury structure
Combined model	0.073	0.022	1.42	0.023	Combined predictors explained only a small proportion of injury-related variability

## Data Availability

The data presented in this study are available on request from the author.

## Supplementary Materials

The following supporting information can be downloaded at https://www.mdpi.com/article/10.3390/jcm15124678/s1, Supplementary Table S1. Correlation structure between morphological, training-related, and injury variables based on Kendall’s Tau coefficients.
