# Supplementary material for: Body Composition Architecture and Injury Topology in Physically Active Young Adults: A Tanglegram-Based Cophylogenetic Approach
Source: J Clin Med. 2026 Jun 16;15(12):4678. doi: 10.3390/jcm15124678 (PMC13301194; doi:10.3390/jcm15124678)
Supplement: Supplementary file 1 [file jcm-15-04678-s001.zip › jcm-4369348-supplementary.pdf]

Supplementary Table S1. Correlation structure between morphological, training-related, and injury variables based on Kendall's Tau coefficients.

variable	injury (0-1)	injury (n)	recurrent	other subsequent	trunk	upper limb	lower limb	sprain	fracture	strain	abrasion
BMI	0,09	0,09	0,08	0,09	0,01	0,08	0,08	0,06	0,05	0,05	0,07
FMI	-0,01	0,00	-0,01	0,02	-0,03	-0,01	0,01	0,03	0,04	-0,01	0,03
FFMI	0,12	0,12	0,10	0,10	0,04	0,11	0,09	0,06	0,03	0,07	0,07
SMI	0,16	0,15	0,12	0,15	0,06	0,19	0,12	0,04	0,01	0,14	0,17
BM	0,12	0,11	0,11	0,09	0,00	0,10	0,10	0,05	0,01	0,07	0,06
TBW	0,14	0,11	0,06	0,11	0,03	0,14	0,10	-0,01	-0,05	0,13	0,19
Trunk muscle mass [kg]	0,16	0,15	0,12	0,13	0,02	0,18	0,13	0,05	0,00	0,14	0,16
Right upper limb muscle mass [kg]	0,17	0,15	0,12	0,12	0,02	0,16	0,14	0,07	0,01	0,12	0,13
Left upper limb muscle mass [kg]	0,16	0,15	0,12	0,12	0,02	0,16	0,13	0,06	0,00	0,12	0,13
Right lower limb muscle mass [kg]	0,18	0,16	0,12	0,14	0,03	0,14	0,16	0,06	0,01	0,12	0,16
Left lower limb muscle mass [kg0	0,18	0,16	0,12	0,14	0,02	0,14	0,16	0,06	0,01	0,12	0,16
Upper limbs asymmetry [%]	-0,05	-0,04	-0,03	-0,02	0,01	0,02	-0,06	-0,03	-0,01	-0,01	-0,05
Lower limb asymmetry [%]	0,05	0,05	0,00	0,05	-0,06	0,01	0,06	-0,03	0,03	0,03	0,06
TPA [MET/min/week]	-0,04	-0,04	-0,02	-0,03	0,02	0,01	-0,07	-0,03	-0,07	-0,03	-0,03
TV [$h \cdot week^{-1}$]	-0,01	0,02	0,07	0,02	-0,03	0,05	0,02	-0,01	-0,03	0,04	0,02
TA [years]	0,06	0,04	0,04	-0,02	-0,01	-0,02	0,06	-0,05	-0,07	0,04	0,05

Abbreviations: BMI - body mass index; FMI - fat mass index; FFMI – free fat mass index, SMI - skeletal muscle mass index; BM – bone mass, TBW – total body water, TPA – total physical activity calculated based on the International Physical Activity Question-naire (IPAQ); TV – training weekly volume (expressed in hours per week); TA – training age (experience expressed in years); SD – standard deviation; CI – confidence interval. Statistically significant values are highlighted in bold.
